# Temporal trend analysis of acute hepatitis B virus infection in China, 1990–2019

**DOI:** 10.1017/S095026882400044X

**Published:** 2024-03-12

**Authors:** Ying Han, Yuansheng Li, Shuyuan Wang, Jialu Chen, Junhui Zhang

**Affiliations:** Department of Epidemiology and Health Statistics, School of Public Health, Southwest Medical University, Luzhou, P. R. China

**Keywords:** acute hepatitis B virus infection, age–period–cohort model, Bayesian age–period–cohort model, joinpoint regression model, temporal trend

## Abstract

China faces challenges in meeting the World Health Organization (WHO)’s target of reducing hepatitis B virus (HBV) infections by 95% using 2015 as the baseline. Using Global Burden of Disease (GBD) 2019 data, joinpoint regression models were used to analyse the temporal trends in the crude incidence rates (CIRs) and age-standardized incidence rates (ASIRs) of acute HBV (AHBV) infections in China from 1990 to 2019. The age–period–cohort model was used to estimate the effects of age, period, and birth cohort on AHBV infection risk, while the Bayesian age–period–cohort (BAPC) model was applied to predict the annual number and ASIRs of AHBV infections in China through 2030. The joinpoint regression model revealed that CIRs and ASIRs decreased from 1990 to 2019, with a faster decline occurring among males and females younger than 20 years. According to the age–period–cohort model, age effects showed a steep increase followed by a gradual decline, whereas period effects showed a linear decline, and cohort effects showed a gradual rise followed by a rapid decline. The number of cases of AHBV infections in China was predicted to decline until 2030, but it is unlikely to meet the WHO’s target. These findings provide scientific support and guidance for hepatitis B prevention and control.

## Introduction

Acute hepatitis B virus (AHBV) infection is an infectious disease caused by HBV, which is primarily transmitted through blood, mother-to-child transmission, and sexual contact. If the initial infection lasts longer than 6 months, chronic HBV infection can develop, leading to liver cirrhosis, liver cancer, and even death [1]. Although widespread use of hepatitis B vaccines and improvements in hygiene conditions have reduced the incidence of HBV infections in recent years [2], approximately 296 million people worldwide still suffer from HBV infections, with an estimated 820 000 deaths according to the World Health Organization (WHO)’s estimate in 2019. Additionally, 1.5 million new infections occur each year [3]. Therefore, HBV infection continues to be a global public health concern [4, 5]. To effectively combat HBV infection, the WHO proposed the goal of eliminating viral hepatitis as a public health threat by 2030 according to the Global Health Sector Strategy on Viral Hepatitis (2016–2020) [6]. In June 2022, the WHO released an action plan for human immunodeficiency viruses, hepatitis viruses, and sexually transmitted infections for 2022–2030, outlining more specific goals and quantifiable indicators to eliminate HBV [7].

China has the highest number of HBV carriers in the world [8]. However, over the past 30 years, China has adopted a series of effective measures to prevent and control HBV infection, with significant results [9]. One of the most important of these measures is the implementation of a hepatitis B vaccine immunization programme for newborns and infants beginning in 1992; this programme was later included in the national expanded immunization programme and was made free of charge in 2002. This enabled newborns and infants to receive the hepatitis B vaccine as soon as possible, effectively reducing their risk of contracting HBV infection. In addition, the government provides significant funding for the purchase of national immunization vaccines and injectors, as well as the launch of catch-up immunization programmes, all of which play important roles. For those who are already infected, antiviral therapy for chronic hepatitis B is an effective way to control disease progression and reduce the occurrence of complications [10]. Moreover, the Chinese National Program for Control and Prevention of Viral Hepatitis (2017–2020) provided strong support for hepatitis B prevention and control efforts [11]. The implementation of these policies and measures has resulted in a decreasing trend in the national reporting incidence of HBV infection in China, dropping below 1% in 2019. As a result, China has transitioned from a high to a middle prevalence area for HBV infection [12]. However, due to China’s large population and significant regional differences, meeting the WHO’s 2030 target [6] of reducing HBV infections by 95% using 2015 as the baseline remains a formidable challenge [8]. Addressing this challenge requires an in-depth understanding of AHBV infection trends and the effectiveness of various policies.

Current research on AHBV infections in China has primarily employed classical time-series models [13, 14], and a limited number of studies have explored joinpoint regression models [15] and age–period–cohort models [16]. However, these studies often lack detailed segmentation across specific age groups and do not fully explore the policy implications of AHBV infection trends. To address these gaps, this study used 30 years of data (1990–2019) to conduct an extensive analysis using a joinpoint regression model, an age–period–cohort model, and a Bayesian age–period–cohort (BAPC) model. This approach aims to provide more nuanced insights into the temporal trends of AHBV infections and to offer more accurate guidance for the formulation and implementation of hepatitis B prevention and control strategies in China.

## Materials and methods

### Data source

Data on national numbers, crude incidence rates (CIRs), and age-standardized incidence rates (ASIRs) of AHBV infections in China from 1990 to 2019 stratified by gender and age groups (in 5-year increments, from 0–4 to 80–84 years) were obtained from the Global Burden of Disease (GBD) 2019. The methods used in GBD studies have been described in detail in previous research [17]. Notably, the GBD data focused on AHBV infection, defined as the presence of hepatitis B surface antigen (HBsAg) for 6 months or less. For the prediction of the ASIR and the number of cases of AHBV infection, the standard population for each age group was derived from the World Standard Population (World Health Organization 2000–2025 Standard–Standard Populations–Surveillance, Epidemiology, and End Results (SEER) Datasets (cancer.gov)), as well as the Chinese population data from the United Nations World Population Prospects website (https://population.un.org/wpp/Download/Standard/CSV/).

### Joinpoint regression analysis

This study used the joinpoint regression model to analyse the temporal trends in the CIR and ASIR of AHBV infections in China from 1990 to 2019. A log-linear model was employed due to data nonnormality (*p* < 0.01) and small sample size (*n* = 30). The best turning point model was selected using Kim et al.’s method [18]. The grid search method was used to select and test the number of turning points. As the study spanned 30 years, a maximum of five turning points were specified. The annual per cent change (APC) and average APC (AAPC) were estimated using the joinpoint regression model, with an APC greater than zero indicating an increasing trend in the CIR and ASIR of AHBV infections in China, an APC less than zero indicating a decreasing trend, and an APC equal to zero indicating stability. The 95% confidence intervals (CIs) of APC and AAPC were calculated. A nonzero CI indicated a significant trend, while a CI containing zero represented a nonsignificant trend at the 0.05 significance level.

### Age–period–cohort and BAPC models

The age–period–cohort model is a statistical method commonly used in the field of epidemiology. The model is based on the Poisson distribution and decomposes the dependent variable into three dimensions, age, period, and cohort, which are used to analyse the incidence risk of AHBV infection at different ages, periods, and cohorts [19]. The expression of the age–period–cohort model is 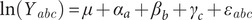 , where 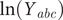 represents the natural logarithm of the dependent variable (CIR of AHBV infection in this study); *μ* is the intercept, representing the baseline incidence risk of age, period, and cohort parameters; 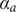 is the age effect for the *a*-th age group; 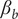 is the period effect for the *b*-th period; 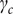 is the cohort effect for the *c*-th cohort; and 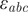 represents the error or residual term [20]. To overcome the identifiability problem caused by collinearity among age, period, and cohort (period minus age equals cohort), we employed a robust approach called the intrinsic estimator age–period–cohort model proposed by Fu et al. [21]. The model estimates coefficients for age, period, and cohort effects that are subsequently transformed into exponential values, which reflect the relative risk (RR) of AHBV infection for a specific age, period, or birth cohort compared to the average level [22]. In the age–period–cohort model, age-specific rates were recoded into continuous 5-year age groups (0–4, …, 80–84), consecutive 5-year periods ranging from 1990 to 2019, and corresponding consecutive 5-year birth cohort groups (1910–1914, …, 2015–2019) to estimate the net effects of age, period, and cohort on the CIR of AHBV infection.

The BAPC model is a Bayesian extension of the age–period–cohort model that employs the integrated nested Laplace approximation method for full Bayesian inference [23]. This model considers age, period, and cohort effects, as well as baseline effects, enabling more accurate predictions and estimates of future trends in specific diseases or health indicators across age, time, and birth cohort populations [24]. This model was used in this study to forecast the annual ASIR and number of AHBV infection cases in China over the next 11 years (2020–2030).

### Software tool

AHBV infection data in China were organized using Excel 2019 software. Joinpoint regression analysis was conducted using the Joinpoint Regression Program 4.8.0.1 (US National Cancer Institute, https://surveillance.cancer.gov/joinpoint/download). Other analyses and data visualizations were performed using R version 4.1.2 (R Foundation for Statistical Computing, Vienna, Austria).

## Results

### Descriptive analyses of AHBV infection rates

**Figure 1.**
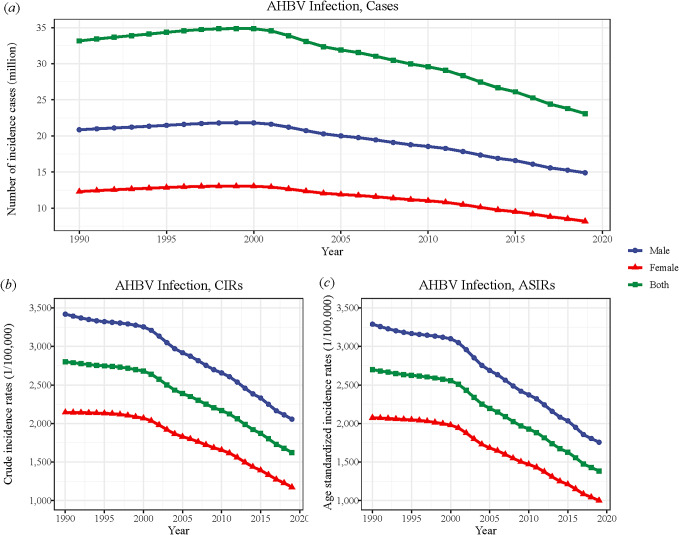
Trends in (a) cases, (b) crude incidence rates (CIRs), and (c) age-standardized incidence rates (ASIRs) of acute hepatitis B virus (AHBV) infections in China from 1990 to 2019.

Over the span from 1990 to 2019, China witnessed a significant decrease in both the number of cases and incidence rates. The total number of cases declined by 30.34%, with females showing a more pronounced reduction of 33.39%, compared to 28.54% in males. Similarly, the CIR decreased by 39.85% in males and 45.24% in females, while the ASIR decreased by 46.57% in males and 51.72% in females over the same period. These changes are comprehensively detailed in Table 1 and Figure 1, which provide a detailed account of changes in cases and incidence rates across genders.

**Table 1.** Changes in cases, crude incidence rates (CIRs), and age-standardized incidence rates (ASIRs) of acute hepatitis B virus (AHBV) infections in China between 1990 and 2019

Measure	Year	Male	Female	Both
Cases (100 000)	1990	208.58	122.98	331.55
	2019	149.04	81.91	230.95
	Percentage change from 1990, %	−28.54	−33.39	−30.34
CIR (per 100 000)	1990	3 418.23	2 144.35	2 801.03
	2019	2 056.22	1 174.29	1 623.71
	Percentage change from 1990, %	−39.85	−45.24	−42.03
ASIR (per 100 000)	1990	3 288.30	2 074.64	2 699.29
	2019	1 756.82	1 001.68	1 383.99
	Percentage change from 1990, %	−46.57	−51.72	−48.73

*Note*: CIRs stand for crude incidence rates of AHBV infections, while ASIRs stand for age-standardized incidence rates. ‘Change’ refers to the per cent change in the number of cases, CIRs, and ASIRs of AHBV infections in China between 1990 and 2019, calculated by subtracting the value in 2019 from the value in 1990.

### AHBV infection trends in China from 1990 to 2019

Joinpoint regression analysis revealed that the CIR (AAPC = −1.86%) and ASIR (AAPC = −2.29%) of AHBV infection in China exhibited significant overall decreasing trends throughout the study period (Table 2 and Supplementary Figure 1).

**Table 2.** Age-specific average annual per cent changes (AAPCs) in crude incidence rates (CIRs) of acute hepatitis B virus (AHBV) infections in China from 1990 to 2019 based on joinpoint regression models, stratified by gender

Age group (year)	AAPC (95% CI), %		
	Male	Female	Both
0–4	−9.26* (−9.90, −8.62)	−9.29* (−9.92, −8.65)	−9.26* (−9.84, −8.68)
5–9	−9.19* (−10.22, −8.15)	−9.24* (−10.30, −8.18)	−9.19* (−10.23, −8.13)
10–14	−8.56* (−9.11, −7.99)	−8.65* (−9.67, −7.63)	−8.58* (−9.14, −8.02)
15–19	−6.53* (−7.05, −6.02)	−6.71* (−7.24, −6.17)	−6.56* (−7.08, −6.04)
20–24	−1.16* (−1.20, −1.12)	−1.18* (−1.26, −1.11)	−1.15* (−1.19, −1.12)
25–29	−1.18* (−1.23, −1.13)	−1.38* (−1.42, −1.34)	−1.26* (−1.30, −1.21)
30–34	−1.22* (−1.28, −1.17)	−1.36* (−1.47, −1.25)	−1.30* (−1.34, −1.26)
35–39	−1.28* (−1.33, −1.23)	−1.41* (−1.48, −1.35)	−1.35* (−1.39, −1.31)
40–44	−1.23* (−1.26, −1.20)	−1.46* (−1.54, −1.38)	−1.33* (−1.41, −1.26)
45–49	−1.23* (−1.31, −1.15)	−1.65* (−1.68, −1.61)	−1.40* (−1.45, −1.36)
50–54	−1.15* (−1.22, −1.08)	−1.65* (−1.87, −1.42)	−1.38* (−1.53, −1.24)
55–59	−1.16* (−1.20, −1.12)	−1.68* (−1.82, −1.53)	−1.38* (−1.43, −1.34)
60–64	−1.14* (−1.23, −1.04)	−1.60* (−1.69, −1.52)	−1.32* (−1.40, −1.24)
65–69	−1.04* (−1.14, −0.94)	−1.75* (−1.91, −1.58)	−1.32* (−1.38, −1.25)
70–74	−1.01* (−1.18, −0.85)	−1.69* (−1.94, −1.45)	−1.24* (−1.42, −1.05)
75–79	−1.00* (−1.06, −0.95)	−1.72* (−1.96, −1.48)	−1.21* (−1.33, −1.09)
80–84	−0.95* (−1.02, −0.88)	−1.70* (−2.10, −1.29)	−1.20* (−1.34, −1.06)
Total	−1.73* (−1.83, −1.64)	−2.04* (−2.14, −1.95)	−1.86* (−1.96, −1.75)
ASIR	−2.14* (−2.29, −1.98)	−2.48* (−2.64, −2.33)	−2.29* (−2.42, −2.16)

*Note*: AAPC represents average annual per cent changes.

* Indicates that AAPC is significantly different from zero at the significance level of 0.05.

For both males and females, notable inflexion points in the CIR and ASIR were observed in 2001, 2004, and 2011, with an additional point for females in 1997. These inflexion points highlight periods of accelerated decline. It was observed that the rate of decline was more rapid for females than for males during this period (Table 3).

**Table 3.** Trends in crude incidence rates (CIRs) and age-standardized incidence rates (ASIRs) of acute hepatitis B virus (AHBV) infections in China from 1990 to 2019 based on joinpoint regression models

	CIR		ASIR	
Gender	Duration	APC (95% CI), %	Duration	APC (95% CI), %
Male	1990–2001	−0.49* (−0.55, −0.43)	1990–2001	−0.58* (−0.68, −0.49)
	2001–2004	−2.74* (−3.59, −1.88)	2001–2004	−3.53* (−4.87, −2.17)
	2004–2011	−1.83* (−1.98, −1.69)	2004–2011	−2.44* (−2.67, −2.21)
	2011–2019	−2.96* (−3.05, −2.86)	2011–2019	−3.45* (−3.60, −3.30)
Female	1990–1997	−0.14* (−0.25, −0.04)	1990–1997	−0.28* (−0.45, −0.11)
	1997–2001	−1.00* (−1.39, −0.62)	1997–2001	−1.04* (−1.67, −0.41)
	2001–2004	−2.92* (−3.67, −2.16)	2001–2004	−3.94* (−5.16, −2.72)
	2004–2011	−1.99* (−2.12, −1.86)	2004–2011	−2.66* (−2.87, −2.45)
	2011–2019	−3.91* (−3.99, −3.83)	2011–2019	−4.38* (−4.52, −4.25)
Both	1990–1998	−0.34* (−0.42, −0.26)	1990–2001	−0.57* (−0.66, −0.49)
	1998–2001	−0.92* (−1.65, −0.19)	2001–2004	−3.87* (−5.04, −2.68)
	2001–2004	−2.73* (−3.45, −2.01)	2004–2011	−2.53* (−2.73, −2.33)
	2004–2011	−1.91* (−2.03, −1.79)	2011–2019	−3.81* (−3.94, −3.68)
	2011–2019	−3.32* (−3.39, −3.24)	NA	NA

*Note*: CIRs stand for crude incidence rates of AHBV infections; ASIRs stand for age-standardized incidence rates; and APC stands for annual per cent change. NA stands for not applicable.

* Indicates that APC is significantly different from zero at a significance level of 0.05.

The most significant decline in the CIR was noted in the 0–4 age group for both genders, with an AAPC of approximately −9.3%. This age group experienced a notably faster decrease in infection rates compared to the oldest age group (80–84 years) (Table 2). Among all 17 age groups, the age group under 20 for both genders exhibited the fastest decline in CIRs and ASIRs (Supplementary Tables 1 and 2 and Supplementary Figures 2 and 3). Notably, the 0–4 age group exhibited a marked downward trend during 2002–2005, followed by significant declines in the 5–9 age group during 2007–2010, the 10–14 age group during 2011–2015, and the 15–19 age group during 2015–2019 (Supplementary Table 3 and Supplementary Figure 4).

### Age–period–cohort analysis

The age–period–cohort analysis revealed that after controlling for the effects of period and cohort, the age-specific RR of AHBV infection for both genders showed a trend towards a rapid increase followed by a slow decline with advancing age. The highest and lowest incidence risks of AHBV infections occurred at ages 25–39 years (RR 1.37) and 0–9 years (RR 0.52), respectively, with the highest risk being 2.63 times greater than the lowest risk. Notably, the age-specific RRs for both genders were greater than 1 and were significantly different for the 20–69 and 20–64 age groups.

For the period effect, after controlling for the effects of age and birth cohort, the incidence risk of AHBV infections showed a decreasing trend across all six periods. The period effect RR decreased from 1.27 to 0.75 for males and from 1.31 to 0.70 for females, decreasing by 40.94% in males and 46.56% in females between 1990–1994 and 2015–2019.

**Figure 2.**
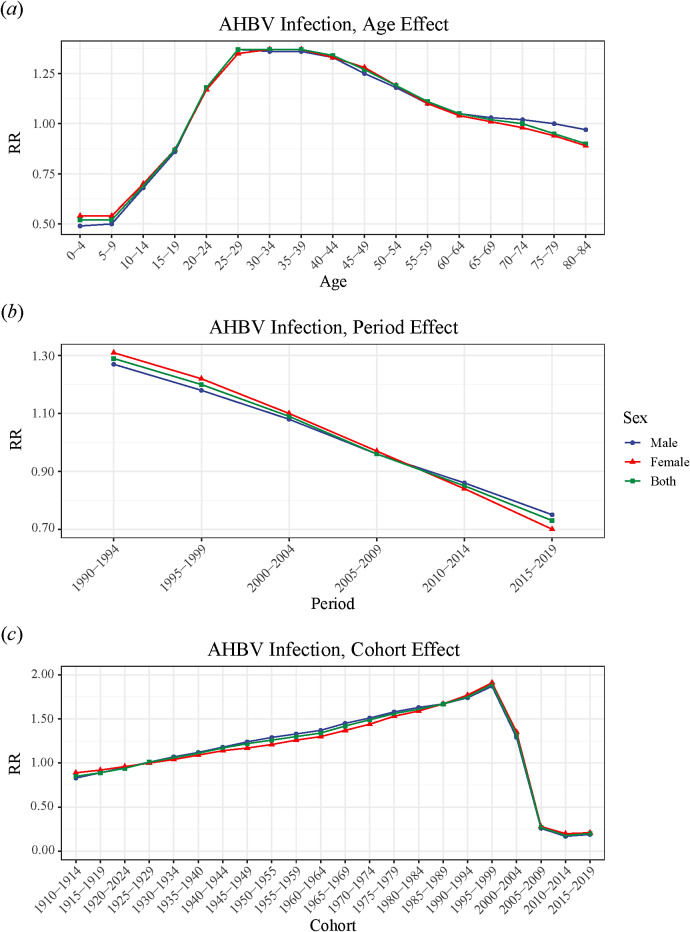
Relative risks (RRs) for the (a) age, (b) period, and (c) cohort effects on the crude incidence rates (CIRs) of acute hepatitis B virus (AHBV) infection in China.

After controlling for age and period effects, the cohort effect RR showed a slow upward trend followed by a rapid downward trend from earlier to later birth cohorts for both genders. A slow increase was observed from the 1910–1914 cohort to the 1995–1999 cohort, while a rapid decrease was observed from the 1995–1999 cohort to the 2005–2009 cohort. In addition, the highest incidence risks were observed for males (RR 1.87) and females (RR 1.91) born during 1995–1999, while the lowest risks were observed for males (RR 0.17) and females (RR 0.20) born during 2010–2014. For males, the highest incidence risk was 11 times greater than the lowest risk, while for females, the highest risk was 9.6 times greater than the lowest risk (Figure 2 and Supplementary Table 4).

### Bayesian age–period–cohort analysis

**Figure 3.**
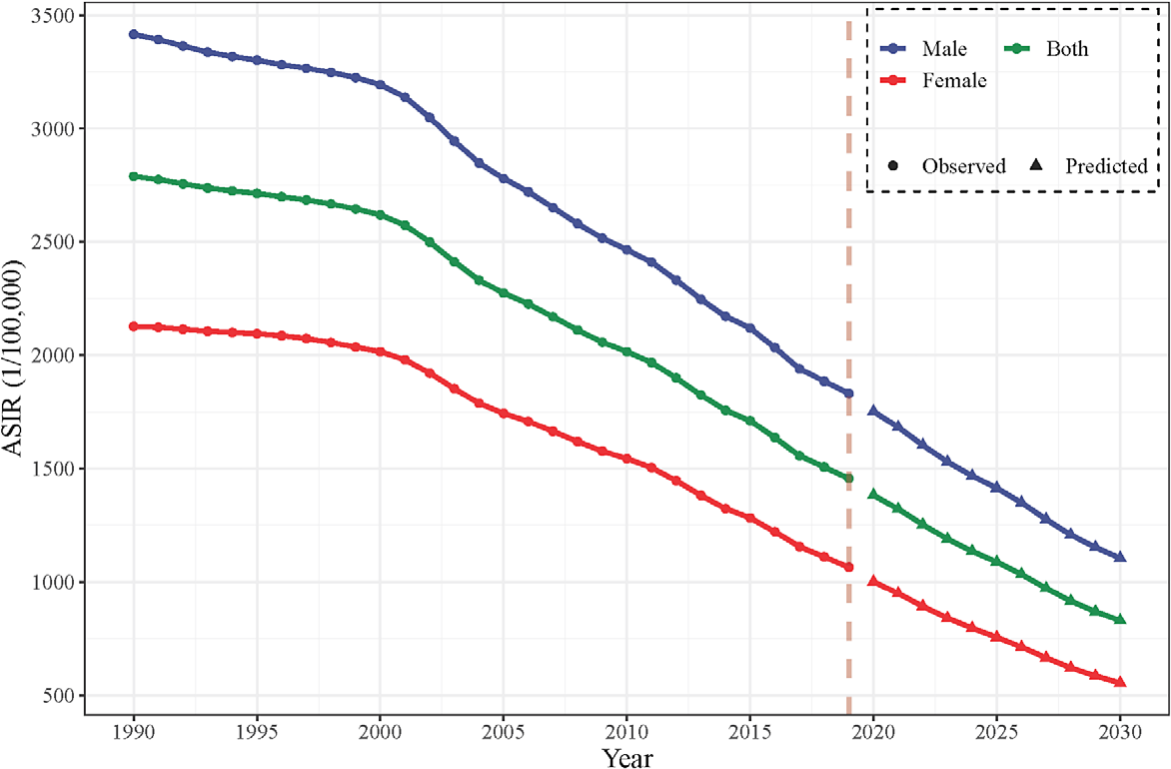
Predictions for annual age-standardized incidence rates (ASIRs) of acute hepatitis B virus (AHBV) infections in China until 2030 based on the Bayesian age–period–cohort (BAPC) model.

**Figure 4.**
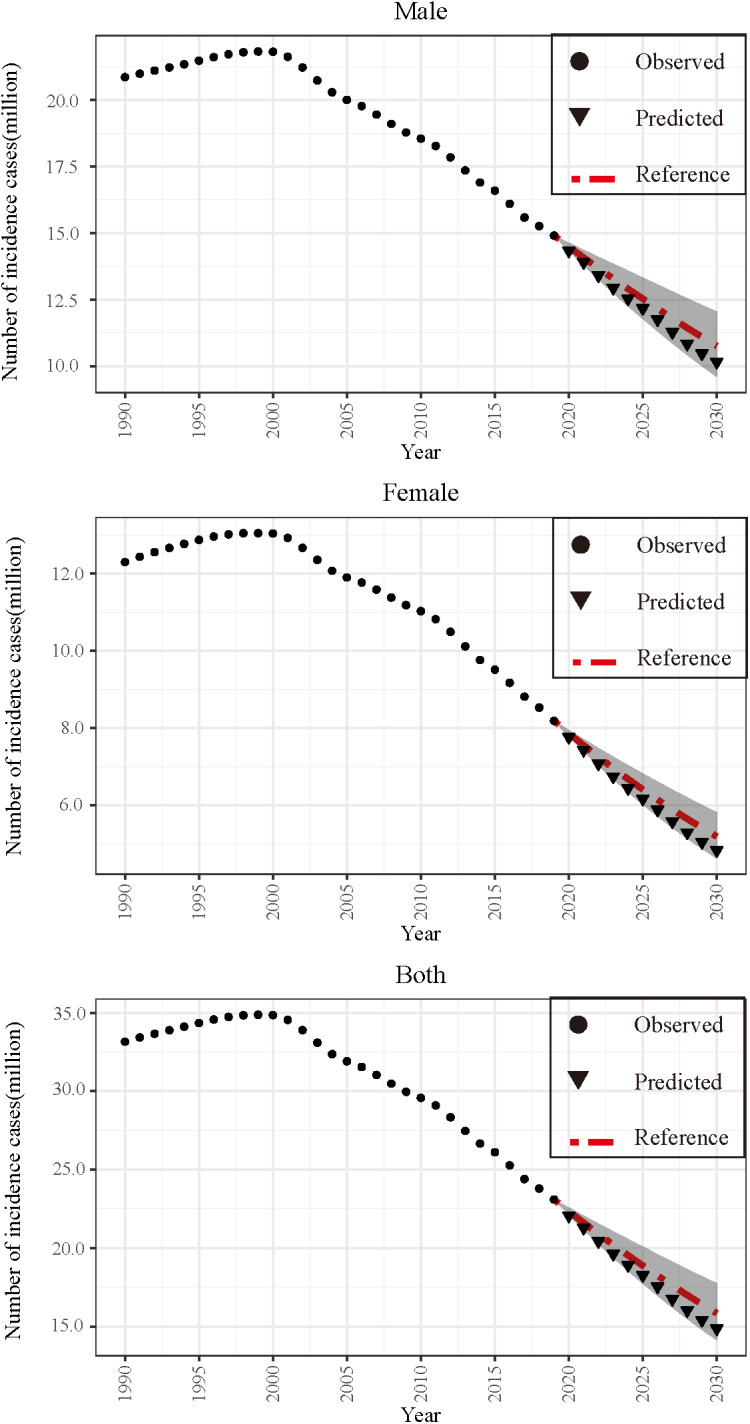
Predictions for the annual number of acute hepatitis B virus (AHBV) infection cases in China until 2030 based on the Bayesian age–period–cohort (BAPC) model, stratified by gender.

The BAPC model forecasts a continued rapid decline in the ASIRs of AHBV infections for both genders from 2020 to 2030 (Figure 3 and Supplementary Table 5). This period is crucial for assessing progress towards the WHO 2016 goal of reducing HBV infections by 95% using 2015 as the baseline year. In addition, the projected decrease in AHBV infection cases by 2030 is substantial, with an expected decrease of 38.80% for males and 49.12% for females (Figure 4 and Supplementary Table 6). Notably, the predictions for AHBV infection cases were based on a linear decline from 2011 to 2019, with an APC of −3.32%, as identified via joinpoint regression analysis. Despite these promising trends, the prediction results suggest that under an optimistic scenario (APC decrease of 1% to −4.32%), China is unlikely to meet the WHO’s 95% reduction target. The pessimistic scenario (APC increase of 1% to −2.32%) presented an even lower likelihood of reaching this target by 2030 (Figure 4 and Supplementary Table 7).

## Discussion

This study reported changes in the number of cases of AHBV infection, CIRs, and ASIRs in different gender and age groups from 1990 to 2019 in China. These findings revealed a significant decrease in all three indicators of AHBV infections in China over the last three decades, which is consistent with the findings of Su et al. [25]. These findings suggest that China’s comprehensive prevention and control measures, mainly based on hepatitis B vaccination, have been effective at reducing the incidence of AHBV infections over the past 30 years. However, due to China’s large population, the absolute number of cases of AHBV infection remains enormous, with significant differences between age groups. Therefore, implementing targeted prevention measures and health education for different populations is critical.

In this study, the age effect in the age–period–cohort model reflects the physiological and social role changes experienced by individuals at different stages of their lives, reflecting the impact of population structure changes on the incidence risk of AHBV infections. The findings revealed that the incidence risk of AHBV infection in both genders was lowest in the 0–9 age group, which is consistent with the findings of previous research [26]. These findings indicate that China’s comprehensive immunization strategy for neonates, combined with a mother-to-child prevention programme and ‘catch-up’ immunization policy for children under the age of 15, has had significant prevention and control effects [27]. The incidence risk of AHBV infection in both genders was highest in the 25–39 age group, with a trend towards a rapid increase followed by a slow decline. This may be due to several reasons. First, the 25–39 age group is sexually active, which increases the risk of AHBV infection through sexual transmission. Second, China included the hepatitis B vaccine in its vaccination programme (vaccine-free) in 2002, but the programme prioritized neonates and children, while the 25–39 age group may have missed some vaccination opportunities [28]. In addition, the incidence risk of AHBV infection in the 60–84 age group decreased, possibly due to factors such as developing immunity [29], a decreased chance of infection, and mild self-limiting infections in the elderly [30].

**Figure 5.**
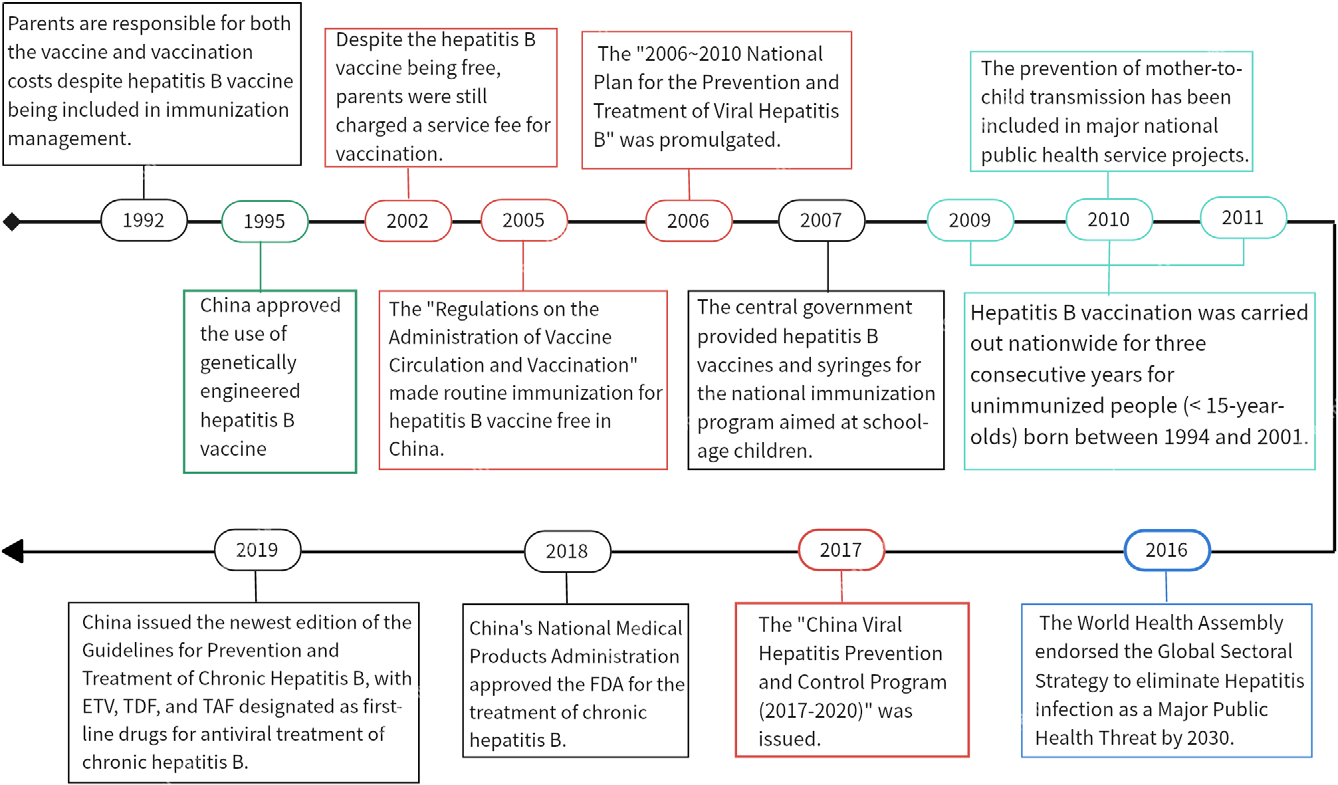
Relevant policies and regulations for preventing and controlling hepatitis B virus infection in China from 1990 to 2019.

The age–period–cohort model’s period effect reflects the impact of changes in related social policies and economic and medical environments during a certain period of time on AHBV infection across all age groups. This study revealed that the incidence of AHBV infection in both genders decreased almost linearly over time. Similar downward trends were observed with joinpoint regression models, and the CIR and ASIR of AHBV infection in China decreased most rapidly from 2011 to 2019. Turning points were observed in 1997, 2001, 2004, and 2011. This may be related to the Chinese government’s comprehensive prevention and control policies for hepatitis B vaccination (free vaccination, supplementary vaccination, drug treatment, etc.), which have been implemented since 1992 [31]. For example, the Chinese State Council issued the ‘Notice on Strengthening Hepatitis B Vaccination’ in 1997, proposing free hepatitis B vaccination for neonates. In 2001, the government called for hepatitis B prevention and treatment. Since 2002, the hepatitis B vaccine has been provided free of charge, but service fees (vaccination fees charged to parents) have continued to be charged. In 2005, routine immunization with the hepatitis B vaccine became free [32], and the ‘National Program for Hepatitis Prevention and Control (2006–2010) [33]’ was introduced in 2006. Since 2007, the hepatitis B vaccine and injectors included in the National Immunization Program for Eligible Children have been fully funded by the central government [34]. In addition, from 2009 to 2011, China provided continuous 3-year hepatitis B vaccination services for unimmunized people (those under the age of 15), vaccinating more than 68 million people [10]. Other policies from various years are depicted in Figure 5. This series of comprehensive measures has ensured the implementation of hepatitis B epidemic prevention and control work, resulting in a significant downward trend in the HBsAg positivity rate in the general Chinese population [12, 35].

The age–period–cohort model’s cohort effect reflects the impact of social change or similar factors on AHBV infection among a group of people who share the same birth period due to their common exposure level. After controlling for age and period effects, the cohort effect RR for both genders showed a slow increase followed by a rapid decrease. This trend may be attributed to several factors. First, prior to 1910–1995, there were relatively few policies for hepatitis B prevention and control and a lack of public health awareness [36]. Second, before 1980, the hepatitis B vaccine had not yet been developed in China, which resulted in a lack of effective preventive measures. Third, although the Chinese government included the hepatitis B vaccine in its routine immunization schedule in 1992, the cost of vaccines and inoculation was borne by parents, resulting in a low vaccination rate before this time [37]. Additionally, since the reform and opening-up policy in 1978, China’s economy has made significant progress, and massive population migration has increased the risk of AHBV infection [38]. However, after 1999, the government implemented a number of policies to improve hepatitis B prevention and control [12], resulting in a significantly greater vaccination rate among individuals born after 1999 and a marked reduction in the risk of AHBV infection.

According to the BAPC model, the ASIRs for both genders in China will continue to decrease rapidly over the next 11 years (2020–2030). However, even with this decline, the number of cases of AHBV infection may not decrease by more than 95% until 2030, falling short of meeting the WHO’s target. Therefore, additional measures must be taken to strengthen the prevention and control of AHBV infection.

## Suggestions

Based on our findings, we propose the following comprehensive prevention and control measures for HBV infection to meet the WHO 2030 target. First, we recommend strengthening routine hepatitis B vaccination and improving coverage among key populations. Second, we recommend strengthening the scientific management and standardized treatment of patients infected with HBV. Third, for the childbearing age group 25–39, we recommend strengthening education on HBV infection prevention and control knowledge in relevant medical institutions, in addition to implementing mother-to-child transmission prevention measures, to improve the timely treatment rate of pregnant women and reduce the risk of mother-to-child transmission [39]. Finally, we recommend regular monitoring and analysis of the epidemiological characteristics of hepatitis B, as well as timely adjustment of prevention and control strategies based on epidemic dynamics.

## Advantages

This study has the following advantages. First, our study has a longer time span and richer data. Compared to most previous studies that covered only a short period of time [40, 41], this study analysed the temporal trends of HBV infection in China over a 30-year period, making the results more representative and reliable. Second, in the analysis using joinpoint regression and age–period–cohort models, we adopted a more precise age group classification method (divided into 5-year intervals). This method is more effective at identifying the HBV infection status and trend changes in different age groups than similar studies with broader age categories (0–14, 15–49, 50–69, and ≥70) [26]. Third, we conducted a comprehensive review of China’s HBV infection prevention and control policies, which highlights a key innovation in our research. With the joinpoint regression model and APC model analysis, we focused on exploring the temporal alignment between the trends in AHBV infection identified by these models and the implementation timeline of China’s prevention and control policies and measures. Finally, we also made both optimistic and pessimistic predictions for the number of AHBV infections, providing valuable insights for public health policy decisions and planning.

## Limitations

The limitations of this study should be acknowledged before interpreting the results. First, the data used in our study came from acute hepatitis B data in the GBD 2019 study, which had varying sources and potential compatibility issues that may lead to bias [14, 25]. Second, the age–period–cohort analysis using the IE method is classified as an ecological study, limiting our ability to draw causal inferences. Finally, our predictions were made using a BAPC model that modelled changes in age, period, and cohort effects based on current trends. This approach did not account for any potential future advancements in treatments or diagnoses.

## Conclusion

This study analysed the temporal trends of AHBV infections in China from 1990 to 2019. The joinpoint regression model results revealed that CIRs and ASIRs decreased from 1990 to 2019, with a faster decline in males and females under the age of 20. The age–period–cohort model revealed that age-specific RRs initially increased rapidly but gradually declined with age after controlling for time periods and birth cohorts. Period-specific RRs showed a downward trend over time after controlling for age and birth cohort, while cohort-specific RRs showed a slow increase followed by a rapid decrease after controlling for age and time period effects. The BAPC model predicted that the ASIRs of both genders will continue to decrease rapidly over the next 11 years (2020–2030), falling short of meeting the WHO target of reducing HBV infections by 95% using 2015 as the baseline. These findings indicate that China has made significant progress in the prevention and control of AHBV infections over the past 30 years, but there are still some gaps. Therefore, to further prevent and control AHBV infections in China, targeted measures such as ensuring adequate vaccination coverage, treating infected pregnant women in a timely manner, and monitoring HBV incidence should be implemented. Improving accessibility to anti-HBV drugs is also vital for early diagnosis and treatment.

## Supporting information

Han et al. supplementary material Han et al. supplementary material

## Data Availability

The data are publicly available. The datasets are available from the corresponding author upon reasonable request via email or via the following websites: Institute for Health Metrics and Evaluation (https://www.healthdata.org/), https://www.cancer.gov/, https://population.un.org/wpp/Download/Standard/CVS/.

## Supplementary material

For supplementary material accompanying this paper visit http://doi.org/10.1017/S095026882400044X. click here to view supplementary material
