# Supplementary material for: Temporal trend analysis of acute hepatitis B virus infection in China, 1990–2019
Source: Epidemiol Infect. 2024 Mar 12;152:e48. doi: 10.1017/S095026882400044X (PMC10964186; doi:10.1017/S095026882400044X)
Supplement: Han et al. supplementary material [file S095026882400044Xsup001.docx]

**Supplementary materials (Supplementary Table 1-7, Supplementary Figure 1-4)**

**Journal**: Epidemiology and Infection

**Title**: Temporal trend analysis of acute hepatitis B virus infection in China, 1990-2019

**Authors**: Ying Han, Yuansheng Li, Shuyuan Wang, Jialu Chen, Junhui Zhang*

***Supplementary Tables 1-7***

**Supplementary Table 1.** Annual percentage change (APC) for the age-specific crude incidence rate (CIR) of acute hepatitis B virus (AHBV) infections in Chinese males based on joinpoint regression models, 1990-2019

| age-group  (year) | Trend 1 | | Trend 2 | | Trend 3 | | Trend 4 | | Trend 5 | | Trend 6 | |
| --- | --- | --- | --- | --- | --- | --- | --- | --- | --- | --- | --- | --- |
|  | Duration | APC (95% CI), % | Duration | APC (95% CI), % | Duration | APC (95% CI), % | Duration | APC (95% CI), % | Duration | APC (95% CI), % | Duration | APC (95% CI), % |
| 0-4 | 1990-1995 | -3.36* (-4.28, -2.43) | 1995-1999 | 5.33* (3.09, 7.61) | 1999-2002 | -8.65* (-12.49, -4.64) | 2002-2005 | -43.87* (-46.23, -41.40) | 2005-2009 | -17.67* (-19.42, -15.88) | 2009-2019 | -0.74* (-1.07, -0.40) |
| 5-9 | 1990-2004 | -0.15 (-0.49, 0.19) | 2004-2007 | -12.84* (-19.02, -6.20) | 2007-2010 | -40.25* (-44.48, -35.69) | 2010-2014 | -14.92* (-17.99, -11.73) | 2014-2019 | -3.36* (-4.94, -1.76) | NA | NA |
| 10-14 | 1990-2011 | -0.95* (-1.15, -0.75) | 2011-2015 | -35.10* (-37.57, -32.54) | 2015-2019 | -15.29* (-17.34, -13.19) | NA | NA | NA | NA | NA | NA |
| 15-19 | 1990-2015 | -1.20* (-1.44, -0.96) | 2015-2019 | -33.93* (-36.46, -31.29) | NA | NA | NA | NA | NA | NA | NA | NA |
| 20-24 | 1990-1994 | -0.90* (-1.01, -0.79) | 1994-2000 | -0.18* (-0.26, -0.10) | 2000-2005 | -2.58* (-2.69, -2.47) | 2005-2010 | -1.74* (-1.85, -1.63) | 2010-2017 | -0.66* (-0.72, -0.60) | 2017-2019 | -1.25* (-1.59, -0.90) |
| 25-29 | 1990-1998 | -0.45* (-0.49, -0.42) | 1998-2001 | -1.20* (-1.55, -0.84) | 2001-2005 | -2.23* (-2.41, -2.06) | 2005-2010 | -0.96* (-1.07, -0.85) | 2010-2015 | -2.19* (-2.30, -2.08) | 2015-2019 | -0.56* (-0.67, -0.44) |
| 30-34 | 1990-1999 | -0.53* (-0.57, -0.49) | 1999-2006 | -1.15* (-1.22, -1.08) | 2006-2014 | -1.87* (-1.92, -1.81) | 2014-2017 | -0.65* (-1.06, -0.25) | 2017-2019 | -2.85* (-3.24, -2.46) | NA | NA |
| 35-39 | 1990-1997 | -0.75* (-0.79, -0.70) | 1997-2004 | -1.15* (-1.21, -1.10) | 2004-2009 | -1.76* (-1.87, -1.66) | 2009-2014 | -1.47* (-1.58, -1.37) | 2014-2017 | -1.05* (-1.38, -0.72) | 2017-2019 | -2.25* (-2.57, -1.92) |
| 40-44 | 1990-1995 | -0.31* (-0.40, -0.22) | 1995-2000 | -1.41* (-1.54, -1.28) | 2000-2010 | -1.08* (-1.12, -1.04) | 2010-2019 | -1.79* (-1.83, -1.75) | NA | NA | NA | NA |
| 45-49 | 1990-1996 | -0.48* (-0.56, -0.40) | 1996-2005 | -1.50* (-1.56, -1.45) | 2005-2011 | -0.97* (-1.08, -0.85) | 2011-2014 | -1.62* (-2.11, -1.12) | 2014-2017 | -2.19* (-2.68, -1.69) | 2017-2019 | -1.07* (-1.56, -0.57) |
| 50-54 | 1990-1995 | -0.09 (-0.32, 0.14) | 1995-2007 | -1.24* (-1.31, -1.17) | 2007-2014 | -2.18* (-2.35, -2.01) | 2014-2019 | -0.53* (-0.76, -0.30) | NA | NA | NA | NA |
| 55-59 | 1990-1996 | -0.22* (-0.30, -0.15) | 1996-2005 | -1.03* (-1.07, -0.98) | 2005-2010 | -1.50* (-1.64, -1.37) | 2010-2016 | -2.18* (-2.28, -2.08) | 2016-2019 | -0.80* (-1.02, -0.58) | NA | NA |
| 60-64 | 1990-1995 | -0.18* (-0.33, -0.03) | 1995-1999 | -1.34* (-1.67, -1.01) | 1999-2007 | -0.58* (-0.67, -0.49) | 2007-2011 | -1.18* (-1.51, -0.85) | 2011-2014 | -3.53* (-4.17, -2.89) | 2014-2019 | -1.33* (-1.48, -1.19) |
| 65-69 | 1990-1995 | 0.21* (0.05, 0.38) | 1995-2000 | -1.41* (-1.63, -1.18) | 2000-2007 | -0.39* (-0.52, -0.27) | 2007-2010 | -1.13* (-1.85, -0.41) | 2010-2016 | -2.76* (-2.92, -2.60) | 2016-2019 | -0.45* (-0.81, -0.09) |
| 70-74 | 1990-1995 | 0.43* (0.16, 0.70) | 1995-2000 | -1.84* (-2.22, -1.46) | 2000-2006 | -0.12 (-0.39, 0.15) | 2006-2011 | -1.18* (-1.56, -0.79) | 2011-2014 | -2.89* (-4.07, -1.70) | 2014-2019 | -1.38* (-1.65, -1.11) |
| 75-79 | 1990-1995 | 0.34* (0.23, 0.45) | 1995-2000 | -1.57* (-1.73, -1.42) | 2000-2006 | 0.29* (0.17, 0.40) | 2006-2011 | -0.98* (-1.14, -0.82) | 2011-2015 | -3.40* (-3.64, -3.15) | 2015-2019 | -1.49* (-1.64, -1.33) |
| 80-84 | 1990-1995 | 0.85* (0.72, 0.98) | 1995-2000 | -1.35* (-1.52, -1.17) | 2000-2006 | 0.10 (-0.03, 0.23) | 2006-2011 | -0.90* (-1.08, -0.72) | 2011-2015 | -3.69* (-3.97, -3.42) | 2015-2019 | -1.53* (-1.71, -1.35) |

Notes: APC stands for annual percentage change; *Indicates that APC is significantly different from zero at a significance level of 0.05; NA stands for not applicable.

**Supplementary Table 2.** Annual percentage change (APC) for the age-specific crude incidence rate (CIR) of acute hepatitis B virus (AHBV) infections in Chinese females based on joinpoint regression models, 1990-2019

| age-group  (year) | Trend 1 | | Trend 2 | | Trend 3 | | Trend 4 | | Trend 5 | | | Trend 6 | |
| --- | --- | --- | --- | --- | --- | --- | --- | --- | --- | --- | --- | --- | --- |
|  | Duration | APC (95% CI), % | Duration | APC (95% CI), % | Duration | APC (95% CI), % | Duration | APC (95% CI), % | Duration | APC (95% CI), % | | Duration | APC (95% CI), % |
| 0-4 | 1990-1995 | -2.36* (-3.28, -1.43) | 1995-1999 | 4.61* (2.42, 6.85) | 1999-2002 | -8.84* (-12.62, -4.89) | 2002-2005 | -44.04* (-46.36, -41.61) | 2005-2009 | -16.40* (-18.16, -14.61) | | 2009-2019 | -1.50* (-1.83, -1.18) |
| 5-9 | 1990-2004 | 0.03 (-0.33, 0.38) | 2004-2007 | -13.28* (-19.56, -6.49) | 2007-2010 | -39.40* (-43.80, -34.67) | 2010-2014 | -14.58* (-17.74, -11.30) | 2014-2019 | -4.99* (-6.57, -3.37) | | NA | NA |
| 10-14 | 1990-2009 | -0.91* (-1.14, -0.68) | 2009-2012 | -9.85* (-16.59, -2.56) | 2012-2015 | -39.24* (-43.78, -34.33) | 2015-2019 | -14.90* (-16.97, -12.78) | NA | NA | | NA | NA |
| 15-19 | 1990-2015 | -1.27* (-1.52, -1.02) | 2015-2019 | -34.52* (-37.13, -31.81) | NA | NA | NA | NA | NA | NA | | NA | NA |
| 20-24 | 1990-1996 | -0.02 (-0.11, 0.07) | 1996-2000 | -1.01* (-1.27, -0.75) | 2000-2004 | -2.08* (-2.34, -1.82) | 2004-2011 | -1.51* (-1.59, -1.42) | 2011-2014 | -1.85* (-2.37, -1.33) | | 2014-2019 | -1.12* (-1.24, -1.01) |
| 25-29 | 1990-1995 | -0.52* (-0.59, -0.45) | 1995-2000 | -0.22* (-0.32, -0.12) | 2000-2005 | -2.59* (-2.69, -2.50) | 2005-2011 | -1.11* (-1.18, -1.05) | 2011-2014 | -2.30* (-2.60, -2.00) | | 2014-2019 | -1.93* (-2.00, -1.86) |
| 30-34 | 1990-1996 | 0.12 (-0.03, 0.27) | 1996-2001 | -1.11* (-1.38, -0.83) | 2001-2004 | -2.08* (-2.94, -1.21) | 2004-2010 | -1.46* (-1.66, -1.27) | 2010-2019 | -2.17* (-2.25, -2.09) | | NA | NA |
| 35-39 | 1990-1992 | 0.93* (0.38, 1.49) | 1992-1996 | 0.01 (-0.27, 0.28) | 1996-2011 | -1.59* (-1.62, -1.57) | 2011-2016 | -2.55* (-2.72, -2.38) | 2016-2019 | -2.03* (-2.30, -1.76) | | NA | NA |
| 40-44 | 1990-1995 | 0.02 (-0.11, 0.15) | 1995-2003 | -1.59* (-1.67, -1.52) | 2003-2010 | -1.24* (-1.34, -1.15) | 2010-2014 | -2.90* (-3.18, -2.63) | 2014-2017 | -1.26* (-1.82, -0.70) | | 2017-2019 | -2.77* (-3.32, -2.21) |
| 45-49 | 1990-1997 | -0.63* (-0.67, -0.60) | 1997-2001 | -1.22* (-1.36, -1.09) | 2001-2004 | -2.34* (-2.61, -2.07) | 2004-2011 | -1.30* (-1.35, -1.26) | 2011-2017 | -2.53* (-2.59, -2.47) | | 2017-2019 | -3.48* (-3.74, -3.21) |
| 50-54 | 1990-1995 | 0.95* (0.58, 1.32) | 1995-2000 | -2.76* (-3.26, -2.26) | 2000-2009 | -1.07* (-1.24, -0.89) | 2009-2014 | -3.43* (-3.92, -2.93) | 2014-2017 | -1.05 (-2.65, 0.58) | | 2017-2019 | -4.25* (-5.80, -2.67) |
| 55-59 | 1990-1995 | 1.05* (0.81, 1.30) | 1995-2000 | -2.89* (-3.23, -2.56) | 2000-2006 | -0.86* (-1.10, -0.62) | 2006-2011 | -2.07* (-2.40, -1.73) | 2011-2014 | -3.95* (-4.99, -2.89) | | 2014-2019 | -2.35* (-2.59, -2.11) |
| 60-64 | 1990-1995 | 0.76* (0.60, 0.93) | 1995-2000 | -2.36* (-2.58, -2.13) | 2000-2006 | -0.45* (-0.61, -0.28) | 2006-2011 | -1.94* (-2.16, -1.71) | 2011-2015 | -4.12* (-4.48, -3.77) | | 2015-2019 | -2.31* (-2.54, -2.09) |
| 65-69 | 1990-1995 | 0.32* (0.05, 0.60) | 1995-2000 | -2.47* (-2.85, -2.09) | 2000-2007 | -0.46* (-0.67, -0.25) | 2007-2011 | -2.03* (-2.63, -1.42) | 2011-2014 | -4.36* (-5.54, -3.17) | | 2014-2019 | -3.04* (-3.30, -2.77) |
| 70-74 | 1990-1995 | 0.92* (0.50, 1.34) | 1995-2000 | -3.19* (-3.77, -2.61) | 2000-2006 | -0.05 (-0.47, 0.37) | 2006-2011 | -1.52* (-2.11, -0.94) | 2011-2014 | -4.66* (-6.44, -2.85) | | 2014-2019 | -3.07* (-3.48, -2.66) |
| 75-79 | 1990-1995 | 1.00* (0.46, 1.54) | 1995-2000 | -3.30* (-4.03, -2.57) | 2000-2006 | -0.10 (-0.63, 0.44) | 2006-2010 | -1.26* (-2.43, -0.07) | 2010-2019 | -3.57* (-3.78, -3.36) | NA | | NA |
| 80-84 | 1990-1995 | 1.05* (0.35, 1.75) | 1995-2000 | -3.02* (-3.96, -2.06) | 2000-2006 | 0.27 (-0.43, 0.96) | 2006-2011 | -2.06* (-3.02, -1.10) | 2011-2014 | -5.18* (-8.07, -2.20) | 2014-2019 | | -2.89* (-3.56, -2.22) |

Notes: APC stands for annual percentage change; *Indicates that APC is significantly different from zero at a significance level of 0.05; NA stands for not applicable.

**Supplementary Table 3.** Annual percentage change (APC) for the age-specific crude incidence rate (CIR) of acute hepatitis B virus (AHBV) infections for both genders in China based on joinpoint regression models, 1990-2019

| age-group  (year) | Trend 1 | | Trend 2 | | Trend 3 | | Trend 4 | | Trend 5 | | Trend 6 | |
| --- | --- | --- | --- | --- | --- | --- | --- | --- | --- | --- | --- | --- |
|  | Duration | APC (95% CI), % | Duration | APC (95% CI), % | Duration | APC (95% CI), % | Duration | APC (95% CI), % | Duration | APC (95% CI), % | Duration | APC (95% CI), % |
| 0-4 | 1990-1995 | -2.93* (-3.77, -2.09) | 1995-1999 | 5.08* (3.07, 7.14) | 1999-2002 | -8.70* (-12.17, -5.10) | 2002-2005 | -43.92* (-46.04, -41.70) | 2005-2009 | -17.17* (-18.76, -15.55) | 2009-2019 | -1.06* (-1.35, -0.76) |
| 5-9 | 1990-2004 | -0.03 (-0.38, 0.32) | 2004-2007 | -13.00* (-19.23, -6.29) | 2007-2010 | -39.92* (-44.23, -35.29) | 2010-2014 | -14.78* (-17.89, -11.55) | 2014-2019 | -4.00* (-5.58, -2.39) | NA | NA |
| 10-14 | 1990-2011 | -0.93* (-1.13, -0.73) | 2011-2015 | -35.05* (-37.53, -32.47) | 2015-2019 | -15.62* (-17.67, -13.51) | NA | NA | NA | NA | NA | NA |
| 15-19 | 1990-2015 | -1.20* (-1.44, -0.96) | 2015-2019 | -34.06* (-36.61, -31.41) | NA | NA | NA | NA | NA | NA | NA | NA |
| 20-24 | 1990-2000 | -0.47* (-0.48, -0.45) | 2000-2004 | -2.50* (-2.62, -2.39) | 2004-2009 | -1.78* (-1.85, -1.70) | 2009-2014 | -1.13* (-1.20, -1.05) | 2014-2017 | -0.56* (-0.79, -0.33) | 2017-2019 | -1.22* (-1.45, -0.99) |
| 25-29 | 1990-1997 | -0.37* (-0.41, -0.33) | 1997-2000 | -0.75* (-1.06, -0.44) | 2000-2005 | -2.34* (-2.44, -2.25) | 2005-2011 | -1.12* (-1.19, -1.05) | 2011-2015 | -2.33* (-2.49, -2.18) | 2015-2019 | -0.93* (-1.03, -0.84) |
| 30-34 | 1990-1996 | -0.29* (-0.34, -0.24) | 1996-2001 | -0.91* (-1.00, -0.82) | 2001-2010 | -1.56* (-1.59, -1.53) | 2010-2014 | -2.13* (-2.27, -1.99) | 2014-2017 | -1.06* (-1.34, -0.77) | 2017-2019 | -2.79* (-3.07, -2.51) |
| 35-39 | 1990-1996 | -0.32* (-0.40, -0.24) | 1996-2005 | -1.39* (-1.44, -1.34) | 2005-2017 | -1.70* (-1.73, -1.67) | 2017-2019 | -2.07* (-2.54, -1.61) | NA | NA | NA | NA |
| 40-44 | 1990-1995 | -0.29* (-0.40, -0.18) | 1995-2003 | -1.39* (-1.46, -1.33) | 2003-2011 | -1.21* (-1.28, -1.15) | 2011-2014 | -2.47* (-2.95, -1.99) | 2014-2017 | -1.57* (-2.05, -1.08) | 2017-2019 | -2.07* (-2.55, -1.59) |
| 45-49 | 1990-1992 | -0.32 (-0.65, 0.01) | 1992-1996 | -0.66* (-0.83, -0.50) | 1996-2001 | -1.38* (-1.48, -1.27) | 2001-2004 | -1.87* (-2.20, -1.55) | 2004-2011 | -1.16* (-1.22, -1.11) | 2011-2019 | -2.09* (-2.12, -2.05) |
| 50-54 | 1990-1995 | 0.29* (0.06, 0.52) | 1995-1999 | -2.04* (-2.53, -1.54) | 1999-2008 | -1.24* (-1.35, -1.13) | 2008-2014 | -2.61* (-2.83, -2.39) | 2014-2017 | -0.72 (-1.73, 0.29) | 2017-2019 | -2.15* (-3.14, -1.15) |
| 55-59 | 1990-1995 | 0.32* (0.23, 0.42) | 1995-2000 | -1.68* (-1.81, -1.56) | 2000-2006 | -1.07* (-1.16, -0.98) | 2006-2011 | -1.88* (-2.00, -1.75) | 2011-2015 | -2.78* (-2.97, -2.58) | 2015-2019 | -1.56* (-1.69, -1.44) |
| 60-64 | 1990-1995 | 0.21* (0.07, 0.34) | 1995-1999 | -1.77* (-2.07, -1.47) | 1999-2007 | -0.67* (-0.75, -0.59) | 2007-2011 | -1.63* (-1.94, -1.33) | 2011-2015 | -3.57* (-3.87, -3.27) | 2015-2019 | -1.47* (-1.66, -1.27) |
| 65-69 | 1990-1995 | 0.36* (0.23, 0.48) | 1995-2000 | -1.80* (-1.97, -1.63) | 2000-2006 | -0.32* (-0.44, -0.20) | 2006-2010 | -1.20* (-1.47, -0.93) | 2010-2015 | -3.34* (-3.51, -3.18) | 2015-2019 | -1.81* (-1.98, -1.64) |
| 70-74 | 1990-1995 | 0.73* (0.41, 1.05) | 1995-2000 | -2.27* (-2.71, -1.83) | 2000-2006 | -0.04 (-0.35, 0.28) | 2006-2011 | -1.25* (-1.69, -0.81) | 2011-2014 | -3.63* (-4.98, -2.26) | 2014-2019 | -2.09* (-2.40, -1.78) |
| 75-79 | 1990-1995 | 0.71* (0.50, 0.92) | 1995-2000 | -2.23* (-2.51, -1.94) | 2000-2006 | 0.28* (0.08, 0.49) | 2006-2011 | -1.08* (-1.36, -0.79) | 2011-2014 | -3.90* (-4.78, -3.00) | 2014-2019 | -2.38* (-2.58, -2.18) |
| 80-84 | 1990-1995 | 1.03* (0.80, 1.27) | 1995-2000 | -2.17* (-2.49, -1.84) | 2000-2006 | 0.34* (0.10, 0.58) | 2006-2011 | -1.23* (-1.56, -0.90) | 2011-2014 | -4.45* (-5.45, -3.44) | 2014-2019 | -2.23* (-2.46, -2.00) |

Notes: APC stands for annual percentage change; *Indicates that APC is significantly different from zero at a significance level of 0.05; NA stands for not applicable.

**Supplementary Table 4.** Relative risks (RR) for the age effects, period effects, and cohort effects on the crude incidence rate (CIR) of acute hepatitis B virus (AHBV) infections in China, stratified by gender

| Factor | China | | |
| --- | --- | --- | --- |
|  | Male | Female | Both |
|  | RR (LCI, UCI) | RR (LCI, UCI) | RR (LCI, UCI) |
| Age (year) |  |  |  |
| 0-4 | 0.49 (0.48, 0.51) | 0.54 (0.52, 0.56) | 0.52 (0.50, 0.53) |
| 5-9 | 0.50 (0.49, 0.52) | 0.54 (0.52, 0.55) | 0.52 (0.50, 0.53) |
| 10-14 | 0.68 (0.66, 0.70) | 0.70 (0.68, 0.72) | 0.69 (0.67, 0.71) |
| 15-19 | 0.86 (0.83, 0.89) | 0.87 (0.85, 0.90) | 0.87 (0.84, 0.90) |
| 20-24 | 1.18 (1.15, 1.22) | 1.17 (1.13, 1.21) | 1.18 (1.15, 1.22) |
| 25-29 | 1.37 (1.33, 1.41) | 1.35 (1.31, 1.40) | 1.37 (1.33, 1.41) |
| 30-34 | 1.36 (1.32, 1.41) | 1.37 (1.33, 1.42) | 1.37 (1.33, 1.41) |
| 35-39 | 1.36 (1.31, 1.40) | 1.37 (1.32, 1.41) | 1.37 (1.33, 1.41) |
| 40-44 | 1.33 (1.29, 1.37) | 1.33 (1.28, 1.37) | 1.34 (1.29, 1.38) |
| 45-49 | 1.25 (1.21, 1.29) | 1.28 (1.24, 1.32) | 1.27 (1.23, 1.31) |
| 50-54 | 1.18 (1.15, 1.22) | 1.19 (1.15, 1.23) | 1.19 (1.15, 1.23) |
| 55-59 | 1.10 (1.07, 1.14) | 1.10 (1.07, 1.14) | 1.11 (1.07, 1.14) |
| 60-64 | 1.05 (1.02, 1.09) | 1.04 (1.01, 1.07) | 1.05 (1.02, 1.08) |
| 65-69 | 1.03 (1.00, 1.06) | 1.01 (0.98, 1.04) | 1.02 (0.99, 1.05) |
| 70-74 | 1.02 (0.99, 1.05) | 0.98 (0.95, 1.01) | 1.00 (0.97, 1.03) |
| 75-79 | 1.00 (0.96, 1.03) | 0.94 (0.91, 0.97) | 0.95 (0.92, 0.98) |
| 80-84 | 0.97 (0.94, 1.00) | 0.89 (0.86, 0.92) | 0.90 (0.87, 0.93) |
| Period (year) |  |  |  |
| 1990-1994 | 1.27 (1.25, 1.29) | 1.31 (1.29, 1.33) | 1.29 (1.27, 1.31) |
| 1995-1999 | 1.18 (1.16, 1.20) | 1.22 (1.20, 1.24) | 1.20 (1.18, 1.22) |
| 2000-2004 | 1.08 (1.06, 1.10) | 1.10 (1.08, 1.12) | 1.09 (1.07, 1.11) |
| 2005-2009 | 0.96 (0.95, 0.98) | 0.97 (0.95, 0.98) | 0.96 (0.95, 0.98) |
| 2010-2014 | 0.86 (0.84, 0.87) | 0.84 (0.83, 0.86) | 0.85 (0.84, 0.86) |
| 2015-2019 | 0.75 (0.74, 0.76) | 0.70 (0.69, 0.71) | 0.73 (0.72, 0.74) |
| Cohort (year) |  |  |  |
| 1910-1914 | 0.83 (0.78, 0.90) | 0.89 (0.82, 0.95) | 0.85 (0.79, 0.91) |
| 1915-1919 | 0.89 (0.85, 0.94) | 0.92 (0.88, 0.97) | 0.89 (0.85, 0.94) |
| 1920-2024 | 0.95 (0.91, 0.99) | 0.96 (0.92, 1.00) | 0.94 (0.90, 0.98) |
| 1925-1929 | 1.01 (0.98, 1.05) | 1.00 (0.97, 1.04) | 1.01 (0.97, 1.04) |
| 1930-1934 | 1.07 (1.04, 1.11) | 1.04 (1.01, 1.08) | 1.06 (1.03, 1.10) |
| 1935-1940 | 1.12 (1.08, 1.15) | 1.09 (1.05, 1.12) | 1.11 (1.08, 1.14) |
| 1940-1944 | 1.18 (1.14, 1.22) | 1.14 (1.10, 1.17) | 1.17 (1.13, 1.21) |
| 1945-1949 | 1.24 (1.20, 1.28) | 1.17 (1.13, 1.21) | 1.22 (1.18, 1.26) |
| 1950-1955 | 1.29 (1.24, 1.33) | 1.21 (1.17, 1.25) | 1.26 (1.21, 1.30) |
| 1955-1959 | 1.33 (1.29, 1.38) | 1.26 (1.21, 1.30) | 1.30 (1.26, 1.35) |
| 1960-1964 | 1.37 (1.32, 1.42) | 1.30 (1.25, 1.35) | 1.34 (1.30, 1.39) |
| 1965-1969 | 1.45 (1.40, 1.50) | 1.37 (1.33, 1.42) | 1.42 (1.37, 1.47) |
| 1970-1974 | 1.51 (1.46, 1.56) | 1.44 (1.39, 1.50) | 1.49 (1.44, 1.54) |
| 1975-1979 | 1.58 (1.53, 1.64) | 1.53 (1.48, 1.59) | 1.56 (1.51, 1.61) |
| 1980-1984 | 1.63 (1.58, 1.68) | 1.59 (1.54, 1.64) | 1.61 (1.56, 1.66) |
| 1985-1989 | 1.67 (1.62, 1.72) | 1.67 (1.62, 1.72) | 1.67 (1.62, 1.72) |
| 1990-1994 | 1.74 (1.69, 1.79) | 1.77 (1.71, 1.82) | 1.75 (1.70, 1.80) |
| 1995-1999 | 1.87 (1.81, 1.93) | 1.91 (1.85, 1.98) | 1.89 (1.83, 1.95) |
| 2000-2004 | 1.29 (1.24, 1.34) | 1.35 (1.30, 1.40) | 1.32 (1.27, 1.37) |
| 2005-2009 | 0.26 (0.25, 0.27) | 0.28 (0.27, 0.29) | 0.27 (0.26, 0.28) |
| 2010-2014 | 0.17 (0.16, 0.18) | 0.20 (0.19, 0.21) | 0.18 (0.17, 0.19) |
| 2015-2019 | 0.19 (0.18, 0.21) | 0.21 (0.20, 0.23) | 0.20 (0.18, 0.22) |

**Supplementary Table 5.** Predictions for annual age-standardized incidence rates (ASIRs) of acute hepatitis B virus (AHBV) infections in China until 2030 based on the BAPC model, stratified by gender

| Year | China | | |
| --- | --- | --- | --- |
|  | Male | Female | Both |
|  | ASIR (1/100,000) | ASIR (1/100,000) | ASIR (1/100,000) |
| 1990 | 3416.37 | 2127.04 | 2789.15 |
| 1991 | 3392.66 | 2123.39 | 2775.09 |
| 1992 | 3363.90 | 2114.68 | 2755.91 |
| 1993 | 3337.26 | 2106.07 | 2737.80 |
| 1994 | 3318.18 | 2100.41 | 2725.02 |
| 1995 | 3301.96 | 2095.06 | 2713.84 |
| 1996 | 3281.78 | 2086.25 | 2699.31 |
| 1997 | 3266.13 | 2073.95 | 2685.35 |
| 1998 | 3247.89 | 2056.19 | 2667.27 |
| 1999 | 3224.86 | 2035.89 | 2645.46 |
| 2000 | 3193.48 | 2015.19 | 2619.20 |
| 2001 | 3138.66 | 1980.07 | 2573.96 |
| 2002 | 3048.91 | 1921.41 | 2499.27 |
| 2003 | 2945.20 | 1852.58 | 2412.38 |
| 2004 | 2848.51 | 1787.85 | 2331.04 |
| 2005 | 2779.63 | 1743.30 | 2273.9 |
| 2006 | 2721.24 | 1707.78 | 2226.72 |
| 2007 | 2651.29 | 1664.15 | 2169.54 |
| 2008 | 2580.47 | 1619.06 | 2111.12 |
| 2009 | 2516.28 | 1577.02 | 2057.60 |
| 2010 | 2465.72 | 1543.22 | 2015.17 |
| 2011 | 2410.33 | 1504.02 | 1967.80 |
| 2012 | 2331.56 | 1445.99 | 1899.15 |
| 2013 | 2246.66 | 1381.71 | 1824.26 |
| 2014 | 2171.29 | 1322.96 | 1756.95 |
| 2015 | 2119.46 | 1281.69 | 1710.36 |
| 2016 | 2033.06 | 1220.90 | 1636.45 |
| 2017 | 1939.66 | 1154.84 | 1556.17 |
| 2018 | 1884.81 | 1110.68 | 1506.53 |
| 2019 | 1830.91 | 1064.04 | 1456.19 |
| 2020 | 1751.37 | 1000.41 | 1382.17 |
| 2021 | 1682.52 | 948.74 | 1321.28 |
| 2022 | 1602.89 | 892.26 | 1252.49 |
| 2023 | 1529.10 | 840.00 | 1188.74 |
| 2024 | 1467.75 | 795.46 | 1135.15 |
| 2025 | 1413.44 | 755.68 | 1087.46 |
| 2026 | 1349.40 | 712.14 | 1033.03 |
| 2027 | 1275.22 | 664.56 | 971.46 |
| 2028 | 1207.24 | 621.05 | 915.06 |
| 2029 | 1152.02 | 584.78 | 868.71 |
| 2030 | 1105.18 | 553.31 | 828.97 |

**Supplementary Table 6.** Predictions for the annual number of cases of acute Hepatitis B virus (AHBV) infections in China until 2030 based on the BAPC model, stratified by gender

| year | China | | |
| --- | --- | --- | --- |
|  | Male | Female | Both |
|  | Cases (No. ×10^5^) | Cases (No. ×10^5^) | Cases (No. ×10^5^) |
| 1990 | 208.58 | 122.98 | 331.55 |
| 1991 | 209.87 | 124.34 | 334.21 |
| 1992 | 211.07 | 125.55 | 336.61 |
| 1993 | 212.24 | 126.64 | 338.88 |
| 1994 | 213.41 | 127.66 | 341.07 |
| 1995 | 214.75 | 128.70 | 343.45 |
| 1996 | 216.08 | 129.58 | 345.66 |
| 1997 | 217.19 | 130.15 | 347.34 |
| 1998 | 217.95 | 130.43 | 348.38 |
| 1999 | 218.24 | 130.46 | 348.70 |
| 2000 | 218.14 | 130.37 | 348.52 |
| 2001 | 216.25 | 129.22 | 345.48 |
| 2002 | 212.21 | 126.65 | 338.87 |
| 2003 | 207.37 | 123.55 | 330.91 |
| 2004 | 202.90 | 120.72 | 323.62 |
| 2005 | 200.06 | 119.00 | 319.05 |
| 2006 | 197.73 | 117.68 | 315.40 |
| 2007 | 194.52 | 115.84 | 310.36 |
| 2008 | 191.02 | 113.79 | 304.81 |
| 2009 | 187.80 | 111.82 | 299.62 |
| 2010 | 185.48 | 110.28 | 295.77 |
| 2011 | 182.72 | 108.21 | 290.93 |
| 2012 | 178.43 | 104.93 | 283.36 |
| 2013 | 173.53 | 101.16 | 274.69 |
| 2014 | 169.00 | 97.63 | 266.63 |
| 2015 | 165.93 | 95.13 | 261.07 |
| 2016 | 160.98 | 91.75 | 252.72 |
| 2017 | 155.82 | 88.17 | 243.98 |
| 2018 | 152.62 | 85.34 | 237.96 |
| 2019 | 149.04 | 81.91 | 230.95 |
| 2020 | 143.41 | 77.70 | 220.70 |
| 2021 | 139.01 | 74.38 | 212.90 |
| 2022 | 134.06 | 70.83 | 204.32 |
| 2023 | 129.40 | 67.48 | 196.23 |
| 2024 | 125.37 | 64.49 | 189.12 |
| 2025 | 121.71 | 61.77 | 182.64 |
| 2026 | 117.50 | 58.90 | 175.46 |
| 2027 | 112.76 | 55.85 | 167.58 |
| 2028 | 108.38 | 53.02 | 160.30 |
| 2029 | 104.71 | 50.57 | 154.09 |
| 2030 | 101.55 | 48.40 | 148.64 |

**Supplementary Table 7.** Predictions for the number of cases of acute Hepatitis B virus (AHBV) infections in China from 2020 to 2030 based on the APC (2011-2019) trend, a pessimistic estimate of a 1% increase or an optimistic estimate of a 1% decrease annually

| Year | Number of cases (No. ×10^5^) | | |
| --- | --- | --- | --- |
|  | Male (down 1%, up 1%) | Female (down 1%, up 1%) | Both (down 1%, up 1%) |
| 2020 | 144.84(143.35, 146.34) | 78.67(77.85, 79.49) | 223.41(221.10, 225.73) |
| 2021 | 140.74(137.85, 143.65) | 75.56(73.99, 77.14) | 216.10(211.65, 220.59) |
| 2022 | 136.74(132.56, 141.01) | 72.55(70.31, 74.84) | 209.00(202.58, 215.55) |
| 2023 | 132.85(127.46, 138.41) | 69.66(66.80, 72.60) | 202.11(193.88, 210.60) |
| 2024 | 129.03(122.52, 135.82) | 66.85(63.45, 70.40) | 195.39(185.49, 205.70) |
| 2025 | 125.27(117.72, 133.22) | 64.14(60.23, 68.25) | 188.81(177.39, 200.83) |
| 2026 | 121.57(113.07, 130.62) | 61.52(57.18, 66.15) | 182.40(169.59, 196.02) |
| 2027 | 117.93(108.55, 128.01) | 58.99(54.25, 64.08) | 176.12(162.07, 191.24) |
| 2028 | 114.36(104.18, 125.42) | 56.55(51.47, 62.07) | 170.02(154.84, 186.52) |
| 2029 | 110.91(100.00, 122.89) | 54.21(48.82, 60.12) | 164.14(147.93, 181.93) |
| 2030 | 107.59(96.00, 120.43) | 51.97(46.32, 58.24) | 158.49(141.36, 177.48) |

***Supplementary Figures 1-4***


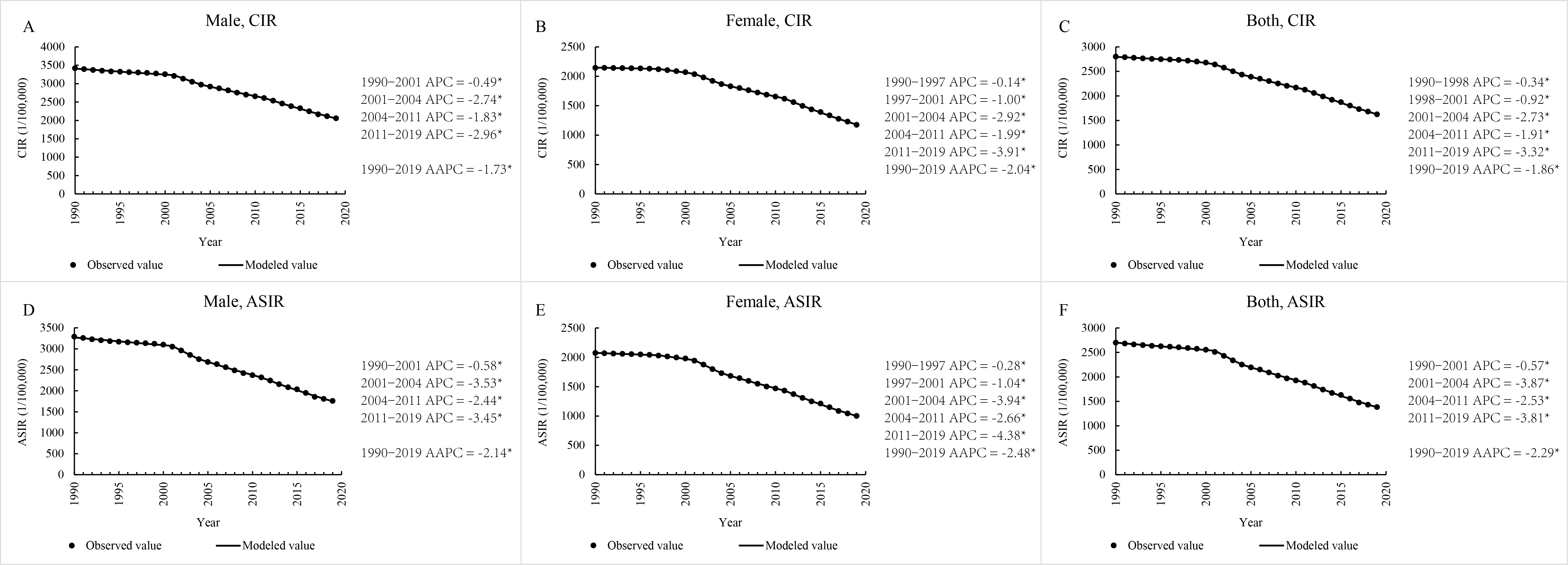


**Supplementary Figure 1.** Crude incidence rate (CIR) and age-standardized incidence rate (ASIR) of acute hepatitis B virus (AHBV) infections in China based on joinpoint regression models from 1990 to 2019, stratified by gender.


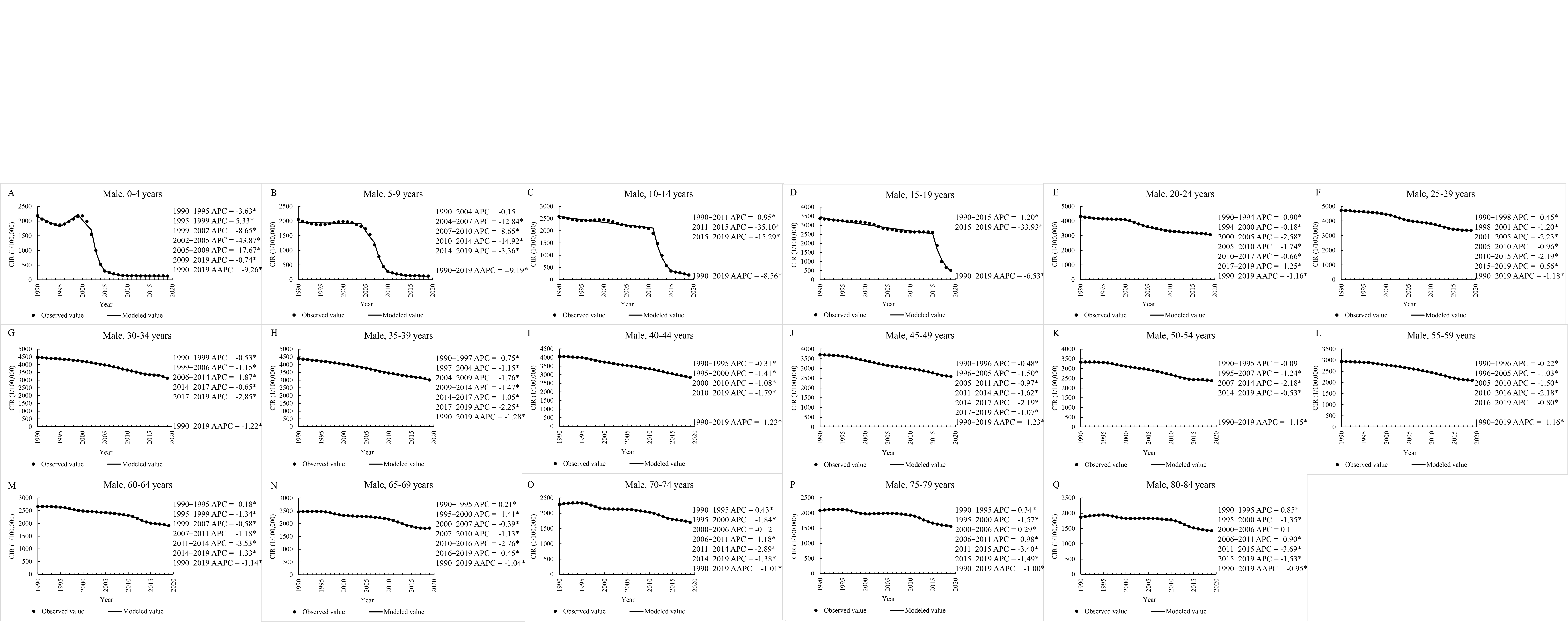


**Supplementary Figure 2.** Annual percentage change (APC) for the age-specific crude incidence rate (CIR) of acute Hepatitis B virus (AHBV) infection in Chinese males based on joinpoint regression models from 1990 to 2019.


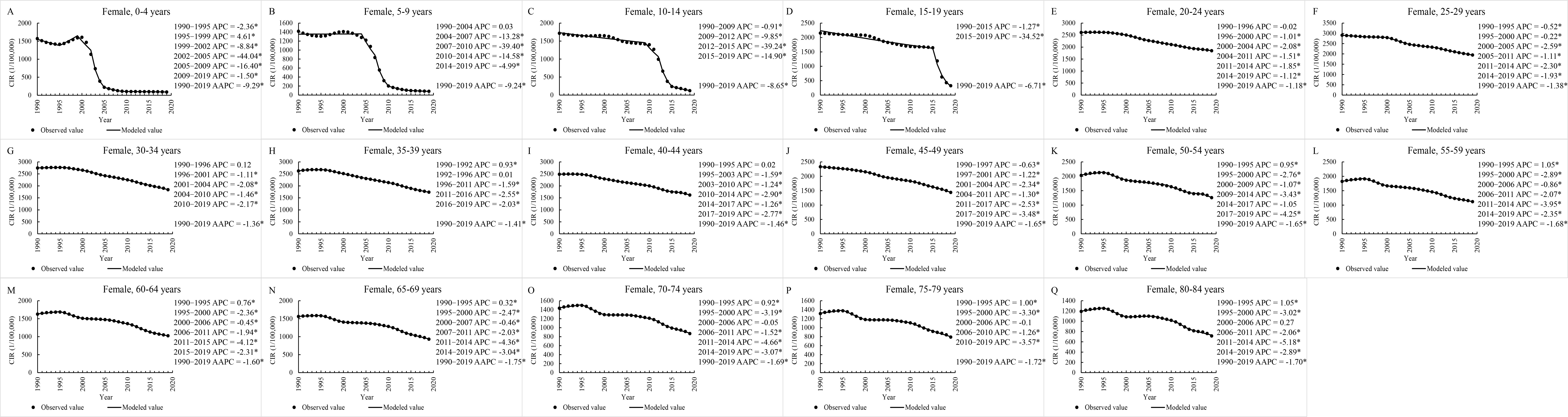


**Supplementary Figure 3.** Annual percentage change (APC) for the age-specific crude incidence rate (CIR) of acute Hepatitis B virus (AHBV) infection in Chinese females based on joinpoint regression models from 1990 to 2019.


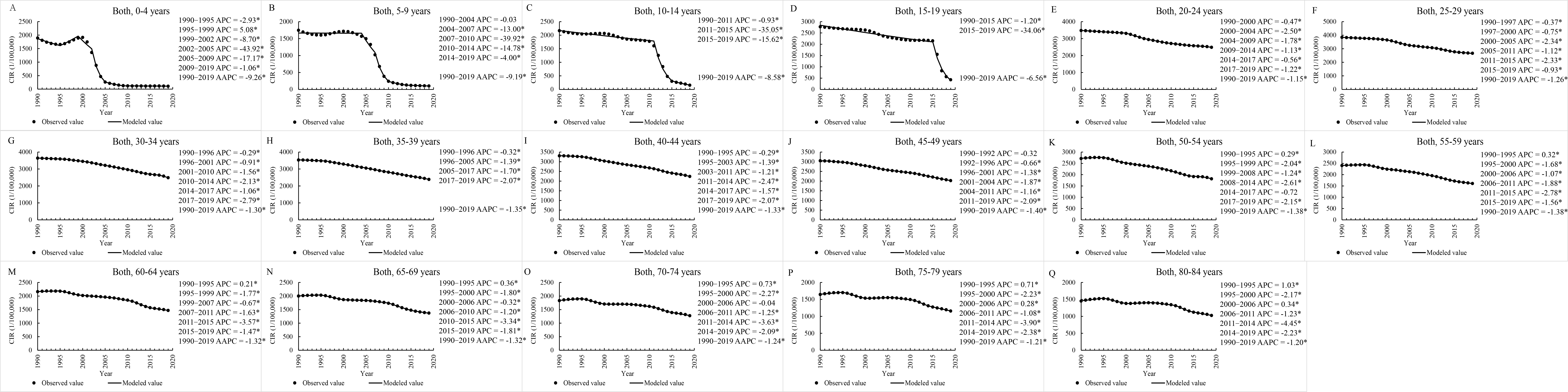


**Supplementary Figure 4.** Annual percentage change (APC) for the age-specific crude incidence rate (CIR) of acute Hepatitis B virus (AHBV) infection for both genders based on joinpoint regression models from 1990 to 2019.
